# Physicochemical and Flavor Characteristics of Maillard Reaction Products from Nile Tilapia Fish Skin Collagen Peptides Induced by Four Reducing Sugars

**DOI:** 10.3390/foods14193453

**Published:** 2025-10-09

**Authors:** Wei Wu, Xilong Wang, Jiayuan Chen, Jingjie Tan, Yu Fu

**Affiliations:** 1 Chongqing Key Laboratory of Herbivore Science, College of Animal Science and Technology, Southwest University, Chongqing 400715, China; weiwu2019@swu.edu.cn (W.W.); sxtytjj318@email.swu.edu.cn (J.T.); 2 College of Food Science, Southwest University, Chongqing 400715, China; wangxilong1117@163.com (X.W.); jiayuan618@outlook.com (J.C.)

**Keywords:** collagen peptides, Maillard reaction, volatile compounds, glucosamine, flavor enhancement

## Abstract

Collagen peptides derived from fish skin may be limited in food applications due to undesirable flavors. To investigate the effects of Maillard reaction modification on their physicochemical and flavor properties, collagen peptides from tilapia skin were prepared via enzymatic hydrolysis, followed by the Maillard reaction with four reducing sugars (xylose, ribose, glucose and glucosamine) through a combined procedure involving simultaneous enzyme inactivation and Maillard reaction at 100 °C. The resultant Maillard reaction products (MRPs) were characterized by analyzing free amino groups, peptide size distribution and color difference, while the reaction progression was monitored using UV absorption and fluorescence spectroscopy. The flavor profile of MRPs was analyzed through quantitative descriptive sensory evaluation and GC-MS coupled with principal component analysis. Among the four reducing sugars tested, glucosamine-induced Maillard reaction products exhibited the most pronounced physicochemical and sensory improvements. Specifically, glucosamine-MRPs showed the greatest reduction in free amino groups (0.69 μmol/L) and a notable decrease in high-molecular-weight peptides (3.31%), accompanied by an increase in low-molecular-weight fractions. Colorimetric analysis revealed a marked color change (Δ*E* = 31.78), and spectral analysis further confirmed intensified UV absorbance and fluorescence intensity in the glucosamine group, indicating advanced reaction progression. Sensory evaluation demonstrated a significant reduction in bitterness and enhancement of umami and saltiness. Moreover, GC-MS analysis revealed that the glucosamine-treated group exhibited the most favorable volatile profile, characterized by an increase in aromatic compounds and a substantial decrease in undesirable odorants. This study provides a theoretical basis for controlling the undesirable flavor of collagen peptides through low-extent Maillard reactions by different reducing sugars.

## 1. Introduction

Collagen is known for its high biocompatibility, excellent bioactivity, and low antigenicity, which makes it widely applicable in the food and cosmetic industries [1,2]. The use of land-based animal collagen has been significantly impacted by outbreaks of diseases in terrestrial animals, such as bovine spongiform encephalopathy (BSE) or mad cow disease, and foot-and-mouth disease [3]. Therefore, fish-derived collagen has a greater potential due to its safety and unique advantages. According to the latest market analysis report, the global collagen market was valued at approximately USD 9.9 billion in 2024 and is projected to reach USD 18.7 billion by 2030, growing at a compound annual growth rate (CAGR) of 11.3%, where Europe secured the largest market share and North America is the fastest-growing market [4]. Furthermore, global collagen peptide market is expected to grow from USD 789.7 million in 2025 to USD 1383.1 million by 2035, at CAGR of 5.7%, while nutritional products are likely to lead the application segment with 42% market share [5]. Fish skin, which occupies about 5–8% of the total weight of fish, is one of the major byproducts during fish processing [6]. It contributes to pollution and landfill accumulation when improperly managed. However, repurposing it into valuable products like collagen offers an economic and sustainable alternative to increase their commercial value as well as to prevent environmental pollution [7,8]. Fish skin is rich in collagen and, after enzymatic hydrolysis, yields fish skin collagen peptides (FSCPs) with various bioactivities, including antioxidant, antihypertensive, and hypoglycemic activities [3]. In addition, FSCPs exhibit potential applications in the fields of food [9], cosmetics [10], and medicine [11]. However, collagen peptides derived from enzymatic hydrolysis normally have unpleasant off-flavors, such as fishy odor and bitterness, which limit their food application [12]. Therefore, how to effectively eliminate the undesirable odor of FSCPs has become an urgent issue to be addressed.

The off-flavor compounds in freshwater fish are primarily formed through two main pathways, including adsorption from the aquaculture environment [13] and spontaneous lipid oxidation [14]. Typical odorants include 2-methylisoborneol (MIB), geosmin (GSM) [15], and various aldehydes and alcohols such as heptanal, octanal, and 1-octen-3-ol [16]. Currently, three approaches are commonly employed to remove off-odors, namely physical methods (e.g., activated carbon adsorption [17]), chemical methods (e.g., Maillard-reaction modification [18]), and biological methods (e.g., yeast fermentation [19]). However, activated carbon treatment frequently leads to the loss of nutritional and flavor-active components. Yeast fermentation, while it is capable of masking or eliminating undesirable odors based on previous studies, lacks specificity and may produce unpredictable metabolic by-products that negatively affect sensory attributes and flavor stability [20].

The Maillard reaction, a non-enzymatic browning reaction between the carbonyl group of reducing sugars and the amino group of amino acids, peptides, or proteins, can generate a variety of pleasant aroma compounds from protein hydrolysates while consuming bitter free amino acids [21,22,23]. Maillard reaction products (MRPs) play a crucial role in the formation of aroma, taste, and color during food processing, and enhance flavor profiles and bioactivities of collagen peptides effectively [23,24]. Previous studies have demonstrated that MRPs may act as free radical scavengers [25], reducing agents [26], and metal chelators [27], owing to their ability to transfer hydrogen atoms and electrons. Nevertheless, the prolonged reaction time at high temperature can result in the formation of undesirable melanoidins and advanced glycation end products (AGEs) [28]. To address this, a controlled Maillard reaction strategy has been proposed by adding reducing sugars during the enzyme inactivation step allows simultaneous Maillard-type glycation at high temperatures [29]. Among reducing sugars, glucosamine, a highly reactive amino sugar, can rapidly induce the Maillard reaction, promote the development of favorable flavor properties, and minimize the formation of AGEs [30]. Additionally, Flavourzyme, which exhibits both endopeptidase and exopeptidase activities, is frequently used to reduce bitterness and enhance overall flavor profile in protein hydrolysates [31]. Based on this rationale, this study employed collagen from tilapia skin as the substrate to explore the effects of Flavourzyme-induced enzymatic hydrolysis coupled with Maillard reaction, induced by four different reducing sugars (xylose, ribose, glucose, and glucosamine), on the physicochemical properties and flavor profiles of FSCPs.

While the Maillard reaction and its impact on flavor profiles have been extensively studied for various protein hydrolysates, the specific application of this reaction to fish skin-derived collagen peptides (FSCPs) remains largely unexplored. Previous studies have focused on the Maillard reaction for improving the flavor of other collagen sources, but no research has specifically addressed the detailed physicochemical and flavor changes induced by Maillard reaction products in FSCPs. Our findings can provide new insights into the potential of FSCPs in food applications, contributing to a deeper understanding of how Maillard reaction products can improve the sensory attributes of fish skin-derived collagen peptides. This study aims to provide a theoretical foundation for improving the flavor of FSCPs through enzymatic hydrolysis coupled with Maillard reaction modification. This study explored the Maillard reaction of fish skin-derived collagen peptides (FSCPs) with four different reducing sugars, specifically investigating their impact on the flavor profile and sensory attributes of FSCPs.

## 2. Materials and Methods

### 2.1. Materials

Tilapia fish skin collagen (protein content, 85.2%) was purchased from Shanghai Xinxi Biotechnology Co., Ltd. (Shanghai, China). Four reducing sugars, including D-xylose (purity > 99%), D-ribose (purity > 99%), glucose (purity > 99%), and glucosamine (purity >99%) were all of food grade and supplied by Shenzhen Yinuo Food Ingredients Co., Ltd. (Shenzhen, China). Flavourzyme (1100 LAPU/g) was obtained from Beijing Solarbio Science & Technology Co., Ltd. (Beijing, China). *N*-acetyl-*L*-cysteine (purity > 99%) and hydrochloric acid (analytical reagent grade) were from Chengdu Chron Chemicals Co., Ltd. (Chengdu, China). *o*-Phthalaldehyde (OPA), and sodium dodecyl sulfate (SDS) were all obtained from Beijing Solarbio Science & Technology Co., Ltd. (Beijing, China). The *L*-leucine standard (purity > 99%) was provided by Adamas Reagent Co., Ltd. (Geneva, Switzerland). All other chemicals used in this study were of analytical grade.

### 2.2. Preparation of FSCPs and MRPs

#### 2.2.1. Preparation of FSCPs

FSCPs were prepared by enzymatic hydrolysis following the method described by Fu et al. [29] with modifications. Briefly, tilapia skin collagen (10 g) was mixed in distilled water (100 mL) to adjust the protein concentration to 10% (*w*/*w*). The mixture was subjected to heat treatment to ensure full solubilization. After cooling, pH was adjusted to 7.0, and Flavourzyme (1100 LAPU/g) was added at the E/S ratio of 1% (*w*/*w*). The hydrolysis was conducted at 50 °C for 6 h based on previous experimental optimization for deodorization performance. After enzymatic hydrolysis with Flavourzyme, degree of hydrolysis (DH) was determined by the OPA assay [32]. The hydrolysate was selected for the subsequent Maillard reaction.

#### 2.2.2. Preparation of MRPs from Different Reducing Sugars

After enzymatic hydrolysis for 6 h, 50 mL aliquots of hydrolysate were withdrawn. Four aliquots of them were mixed with different reducing sugars (xylose, ribose, glucose, and glucosamine) at mass ratio of 5:1 (peptides: sugar, *w*/*w*), which is based on our previous study with a slight modification [29]. The mixtures were heated at 100 °C for 1 h to simultaneously inactivate the enzyme and induce the Maillard reaction. After the reaction, samples were immediately centrifuged at 4 °C (H3–16KR, Hunan Kecheng Instrument Equipment Co., Ltd., Changsha, China). The supernatant was further freeze-dried for further analysis. The remaining 6 h hydrolysate aliquot without added sugar served as the control group. After freeze-drying, sample solution prepared by distilled water at different concentrations for each analysis. Different concentrations were used for further analysis, including 50 mg/mL for color difference and GC-MS, 5 mg/mL for spectral analysis, 20 mg/mL for sensory analysis.

### 2.3. Determination of Free Amino Group

The content of free amino groups was measured by OPA method as described by Fu et al. [33]. The OPA reagent was prepared by mixing 8 mL of 50 mmol/L OPA, 8 mL of 50 mmol/L *N*-acetyl-*L*-cysteine, 4 mL of 200 mg/mL SDS, and 60 mL of 100 mmol/L borate buffer (pH 9.8). Then, 30 μL of each sample was added to 3.6 mL of OPA reagent, and the mixture was incubated in the dark at 25 °C for 10 min. Absorbance was measured at 340 nm using a UV-vis spectrophotometer (UV-6100, Shanghai Yuanxi Instruments Co., Shanghai, China). The calibration curve was established according to Formula (1) using *L*-leucine standard solutions:

y = 0.0602x + 0.0735 (R^2^ = 0.9999) (1)

where x is the mass concentration of *L*-leucine (mg/mL), and y is the absorbance (A).

### 2.4. Determination of Molecular Weight (MW) Distribution

The MW distribution of FSCPs and MRPs was determined by size exclusion chromatography (SEC) using a Phenomenex BioSep™ SEC-S2000 column (300 mm × 4.6 mm) (Torrance, CA, USA) on a high-performance liquid chromatography (HPLC) system (Ultimate 3000, Thermo Fisher Scientific Inc., Waltham, MA, USA), as described by Fu et al. [34]. Data were processed and acquired via Chromeleon 7.0 Chromatography Data System software. Ten microliters of each sample (1 mg/mL, filtered through 0.22 μm membranes) were injected, eluted isocratically using 30% acetonitrile containing 0.1% trifluoroacetic acid at the flow rate of 0.5 mL/min at 25 °C and monitored at the wavelength of 214 nm.

### 2.5. Color Difference Analysis

Color difference was determined using a colorimeter (Chroma Meter CR-400 (Konica Minolta, Tokyo, Japan). The L* (lightness), a* (redness), and b* (yellowness) values were recorded, and the total color difference (Δ*E*) was calculated relative to the control (6 h hydrolysate without reducing sugar) using the following Formula (2):

(2) $$∆E=\sqrt{{∆L}^{*2}+∆a^{*2}+∆b^{*2}}$$

### 2.6. Spectral Analysis by UV and Fluorescence Scanning Spectroscopy

The intensity of Maillard reaction induced by different reducing sugars was quantitatively determined using UV-vis scanning and fluorescence spectroscopy. UV–vis absorption spectra were recorded from 200 to 500 nm using UV–vis spectrophotometer (UV-6100, Shanghai Metash Instruments Co., Ltd., Shanghai, China). Fluorescence spectra were recorded with excitation at 347 nm and emission scanning from 370 to 600 nm using a fluorescence spectrophotometer (F-2500, Hitachi, Ltd., Tokyo, Japan). All MRP samples were diluted to 5 g/L using ultrapure water before analysis.

### 2.7. Sensory Evaluation of MRPs

#### 2.7.1. Panelist Selection and Training

A total of 10 sensory panelists (five males and five females) were selected through a two-step process involving taste recognition and sorting accuracy. Participants provided written informed consent before taking part in the study. Each candidate underwent taste recognition and sorting tests using concentration gradient solutions for five basic tastes. Specifically, the solutions included sucrose at 10, 30, and 50 mmol/L for sweetness; lactic acid at 10, 20, and 30 mmol/L for sourness; sodium chloride at 10, 20, and 30 mmol/L for saltiness; caffeine at 1, 2, and 3 mmol/L for bitterness; and monosodium glutamate (MSG) at 10, 20, and 30 mmol/L for umami. Candidates who demonstrated the highest sorting accuracy across these solutions were selected as the sensory evaluation panelists. Panelists received daily 2 h training sessions focused on recognizing and discriminating the five basic taste modalities. The training duration was determined based on individual performance improvements.

#### 2.7.2. Taste Profile Analysis by Quantitative Descriptive Analysis (QDA)

The taste characteristics of MRPs prepared with different reducing sugars were evaluated using QDA. Five basic taste attributes sourness, sweetness, bitterness, saltiness, and umami were quantitatively assessed by trained panelists using specific methods:

*Establishment of evaluation criteria.* Sensory scoring criteria were established using reference solutions. Midpoint reference samples (assigned a score of 7.5) included 50 mmol/L citric acid, 50 mmol/L sucrose, 1 mmol/L caffeine, 25 mmol/L NaCl, and 3 mmol/L MSG. Upper-limit reference solutions (assigned a score of 15) consisted of 100 mmol/L citric acid, 100 mmol/L sucrose, 2 mmol/L caffeine, 50 mmol/L NaCl, and 6 mmol/L MSG.

*QDA procedure.* During evaluation, each panelist received 1 mL of MRP solution. Samples were presented in randomized order, and panelists were instructed to taste each sample and immediately assess the five taste attributes. All evaluations were conducted under the guidance of a trained moderator to ensure consistency and reduce bias.

*Establishment of scoring scale.* A 15 cm unstructured linear scale was established for sensory intensity scoring [35]. Panelists marked the perceived strength of each attribute along the scale. The distance from the left endpoint to the marked position was measured in centimeters and converted into a numerical score for statistical analysis.

The sensory analysis results obtained from QDA were subjected to statistical analysis using one-way analysis of variance (ANOVA) to evaluate the significant differences in the taste attributes (sourness, sweetness, bitterness, saltiness, and umami) among MRPs prepared with different reducing sugars. Duncan’s multiple comparison test was applied to further identify which specific groups differed significantly (*p* < 0.05).

### 2.8. Volatile Compound Analysis of MRPs by GC–MS

Volatile compounds produced during the Maillard reaction were analyzed using a gas chromatography–mass spectrometry (GC–MS) system (QP2010, Shimadzu Corporation, Kyoto, Japan) equipped with a DB-5MS capillary column (30 m × 0.25 mm, 0.25 μm). The method was adapted from a previous study with modifications [36]. The oven temperature was programmed as follows: initial temperature 40 °C held for 2 min, then ramped to 70 °C at a rate of 5 °C/min, to 120 °C at a rate of 3 °C/min, to 150 °C at a rate of 5 °C/min, to 165 °C at a rate of 2 °C/min, to 210 °C at a rate of 15 °C/min, and finally to 250 °C at a rate of 10 °C/min, held for 5 min. As for MS conditions, the injector and ion source temperatures were both set at 250 °C. Full scan mode was used with a mass range of 35–500 *m*/*z*. Compound identification was performed by comparison with the NIST08 standard mass spectral database, and only matches with a similarity index (SI) > 85% were considered. The peak area normalization method was used for quantitative analysis of the components.

### 2.9. Statistical Analysis

All the experiments were conducted in triplicate. Results are presented as mean ± standard deviation. One-way analysis of variance (ANOVA) and post hoc multiple comparisons were performed using SPSS 22.0 (IBM Corp., Armonk, NY, USA), with Duncan analysis applied to determine significant differences among groups (*p* < 0.05). Graphs were generated using SigmaPlot 14.0 (Systat Software Inc., San Jose, CA, USA) to visualize the experimental results. Principal component analysis (PCA) was carried out using The Unscrambler X 10.4 (CAMO Software, Oslo, Norway) to further interpret and visualize the data.

## 3. Results and Discussion

### 3.1. Changes in Free Amino Group Content of MRPs

**Figure 1:**
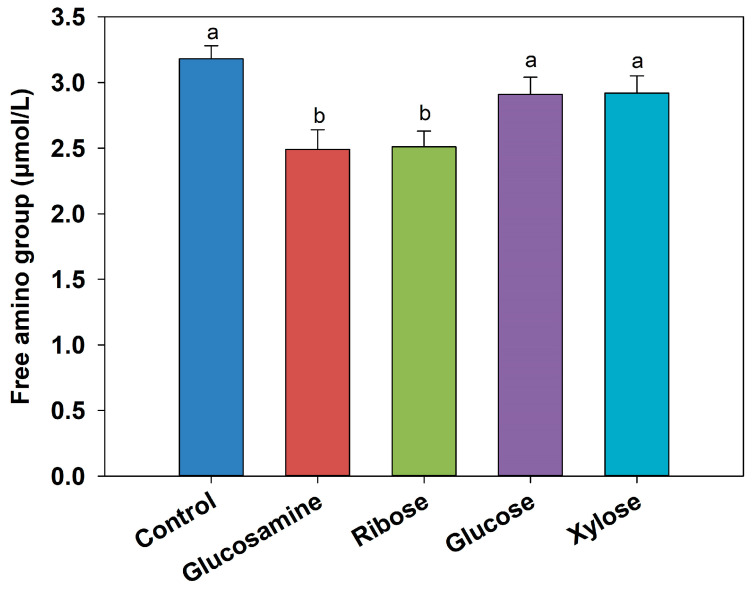
The changes in free amino groups in MRPs from different reducing sugars. Different letters between each group indicate significant differences between treatments (*p* < 0.05).

Since the Maillard reaction consumes free amino groups to generate various intermediate products, the changes in free amino content and MW distribution can reflect the extent of Maillard reaction progression. As shown in Figure 1, FSCPs (DH, 22%) were reacted with four different reducing sugars at 100 °C, and the free amino group concentrations in all the MRPs were lower than that of the control group. Notably, MRPs prepared with ribose and glucosamine showed the most significant decreases, with reductions of 0.67 μmol/L and 0.69 μmol/L, respectively (*p* < 0.05). This fact indicated that more free amino groups are consumed as the Maillard reaction proceeds [37]. The similar findings have been reported in several previous studies. For instance, Sun et al. [38] found that the free amino content in MRPs of xylose and wheat gluten peptides at 100 °C decreased by 25.5%, compared to untreated hydrolysates. Similarly, Qin et al. [39] observed that when sesame protein hydrolysates underwent Maillard reactions with xylose, glucose, and fructose, the free amino acid contents decreased by 71.06%, 59.24%, and 68.66%, respectively.

Although the amino acid composition of FSCPs and their MRPs was not directly analyzed, we acknowledge its importance in understanding the flavor formation process. Previous studies have shown that amino acid composition is a key factor influencing the reactivity with reducing sugars during the Maillard reaction [22]. However, our study primarily focuses on the changes in peptide structure and the resulting flavor characteristics, which are influenced by the overall peptide structure rather than individual amino acids. Hence, this aspect will be further explored in greater detail in our future studies.

### 3.2. Changes in MW Distribution of MRPs

**Figure 2:**
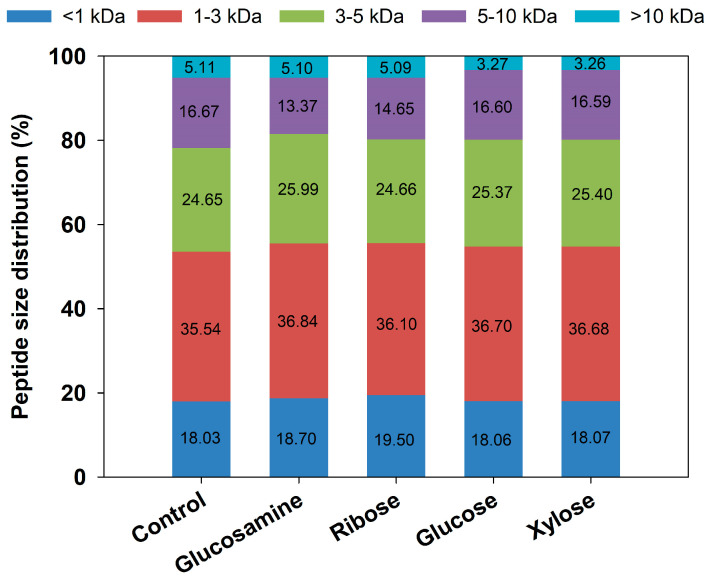
Molecular weight distribution of MRPs from different reducing sugars.

The changes in MW can indicate the degradation and crosslinking of peptides during the Maillard reaction. Figure 2 illustrates the changes in MW distribution of FSCPs during the Maillard reaction. In the control group, the peptide fractions with MW > 10 kDa, 5–10 kDa, and 3–5 kDa accounted for 5.11%, 16.67%, and 24.65%, respectively, while those <3 kDa represented the highest proportion at 53.57%. The abundance of low-MW peptides and amino acids served as a foundation for further Maillard reactions and could also accelerate their progression. After reacting 6 h hydrolysate with the four different reducing sugars, the proportion of high-MW peptides slightly decreased, likely due to heat-induced degradation of high MW peptides. Among them, the most pronounced reduction was observed in the glucosamine group, where the proportion of high-MW components decreased by 3.31%. Conversely, the proportion of low-MW peptides and amino acids increased slightly, with the ribose group showing the largest increase at 1.47%, indicating that the degradation of larger peptides exceeded that of smaller ones. These findings are consistent with the study by Yu et al. [40], in which the Maillard reaction occurred between glucosamine and fish viscera peptides, leading to a significant decrease in the proportion of peptide fraction > 5 kDa, from 38.62% to 8.16%, while those <1 kDa increased from 1.31% to 6.24%.

### 3.3. Color Difference Data of MRPs

The Maillard reaction typically causes browning, and a more intense reaction generally leads to more pronounced color changes [41]. Therefore, color difference analysis can reflect the degree of Maillard reaction among the four reducing sugars. Table 1 presents the colorimetric values (L*, a*, b*, and *ΔE*) of the sample solutions. As the reaction progressed, the L* values of the MRPs decreased, indicating that the hydrolysate without Maillard reaction (control) had the highest brightness. In contrast, the b* values increased upon reaction, suggesting enhanced yellow–blue coloration in the MRPs. The a* values of the glucosamine- and ribose-derived MRPs increased, indicating stronger red–green tones in these samples. The observed color changes were direct consequences of Maillard reaction progression. Herein, a higher positive a* value indicates a shift toward redness, while a higher positive b* value signifies increased yellowness. The significantly higher a* and b* values in the ribose and glucosamine groups, compared to glucose and the control, can be attributed to the superior reactivity of these sugars. Ribose, as a pentose, underwent Maillard reaction much faster than hexoses like glucose and xylose, leading to accelerated formation of red and yellow colored intermediates and melanoidins [42]. Similarly, glucosamine, as an amino sugar, presents a highly reactive carbonyl group that readily condenses with amino groups, promoting rapid browning [30,43]. In contrast, glucose-induced MRPs showed a* and b* values similar to the control, suggesting that the Maillard reaction progressed to a much lesser extent under the given conditions, which is consistent with its relatively lower reactivity.

In addition, the continuous decrease in L* value can be attributed to the formation of brown melanoidins during the Maillard reaction. Due to the accumulation of browning pigments and caramelization products, the MRPs solutions exhibited visible red and yellow hues [44]. Moreover, Δ*E* values of glucosamine and ribose groups were significantly higher than those of other groups, confirming a greater overall color difference and a deeper progression of the Maillard reaction [45]. It is worth noting that caramelization appears to significantly contribute to color development only in fructose–glycine systems. In other sugar systems, such as glucose, glucosamine, and ribose, the impact of caramelization is negligible [42]. Therefore, the browning observed in the MRPs solutions is predominantly due to the Maillard reaction, while the effects of thermal degradation and sugar caramelization are minimal.

### 3.4. Spectral Analysis of MRPs

#### 3.4.1. UV Scanning Spectra of MRPs

**Figure 3:**
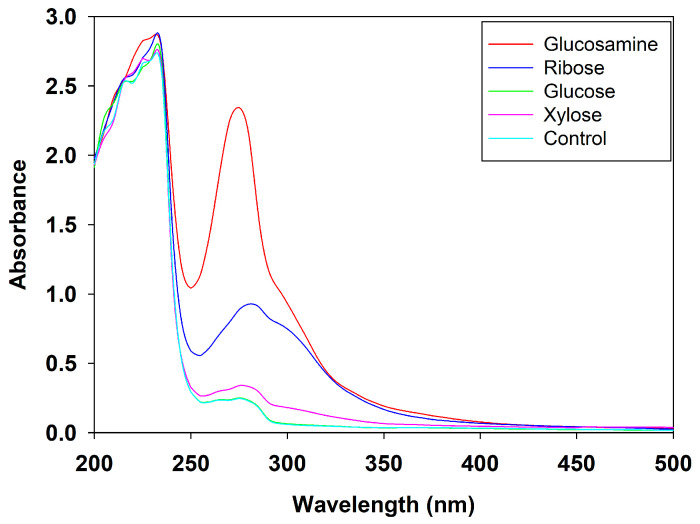
UV scanning spectra of MRPs from different reducing sugars.

UV scanning is a widely used method to monitor and characterize the progress of the Maillard reaction [46,47]. The absorbance at 320 nm typically reflects the formation of intermediate Maillard reaction products, while the absorbance at 420 nm is commonly used to indicate the formation of advanced Maillard reaction products such as melanoidins [29]. As shown in Figure 3, the MRPs from all four reducing sugars exhibited higher absorbance than the control group in the range of 320–420 nm. Notable differences were also observed in the region between 280 and 294 nm, suggesting the formation of certain intermediate compounds during the reaction. Specifically, the peak near 280 nm is associated with the generation of sugar condensation products and the presence of aromatic amino acids [48], while the absorbance at 294 nm corresponds to the formation of dicarbonyl compounds such as ketones and aldehydes during the Maillard reaction [49]. Among all groups, the MRPs derived from glucosamine exhibited the highest absorbance in the region of 280–294 nm, confirming that glucosamine induced the production of more intermediate compounds during the Maillard reaction.

These results were in good agreement with the recent findings of Yu et al. (2024), who also observed a gradual increase in UV absorption for MRPs derived from fish protein hydrolysates and glucosamine, confirming the high reactivity of glucosamine in Maillard reaction [40]. Similarly, Sun et al. (2023) [38] reported that Maillard reactions involving wheat protein hydrolysates and ribose or glucose resulted in similar UV absorbance patterns. In their study, samples containing only heated wheat protein hydrolysates exhibited minimal UV absorption between 240 and 294 nm, and no obvious peaks were detected when glucose or ribose was heated alone [38]. The present results were also consistent with the findings of Fu et al. (2023), where the absorption value at 420 nm represented the extent of browning, and 294 nm indicated the formation of early and intermediate flavor compounds or precursors [50]. These results further supported the fact that UV absorbance in this range is primarily attributable to the formation of Maillard reaction products [51].

#### 3.4.2. Fluorescence Scanning Spectra of MRPs

**Figure 4:**
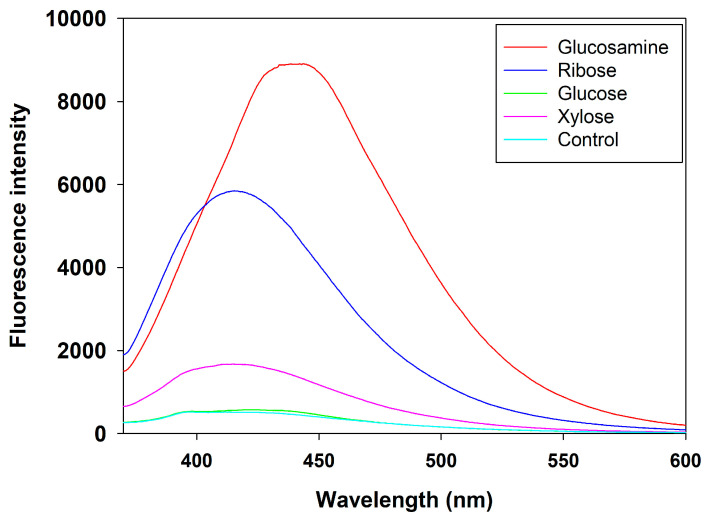
Fluorescence scanning spectra of MRPs from different reducing sugars.

Fluorescence spectroscopy is a valuable technique for analyzing and characterizing MRPs, as it provides insights into the spectral properties, concentration, and intensity of fluorescent compounds generated during the reaction [43]. The fluorescence emission spectra of MRPs derived from different reducing sugars are presented in Figure 4. Overall, compared to the FSCPs without sugar, all four MRPs exhibited significantly higher fluorescence intensity within the wavelength range of 400~550 nm. This enhancement in fluorescence can be attributed to the generation of intermediate Maillard reaction products with intrinsic fluorescent properties [52]. Among the tested sugars, the MRPs derived from glucosamine showed the highest fluorescence intensity in the range of 400–550 nm, indicating the formation of the largest quantity of fluorescent compounds.

These results were consistent with the findings of Yu et al. (2022) [53], who studied MRPs prepared from grass carp collagen peptides and glucose. They observed that the fluorescence intensity of the Maillard reaction products increased progressively with reaction time and was consistently higher than that of the control group, suggesting the continued formation of fluorescent intermediates as the Maillard reaction proceeded. Similarly, this result aligns with the general principle of the Maillard reaction, where increasing reaction time leads to the gradual accumulation of reaction products, as also demonstrated by Li et al. (2023) with xylose and corn zein [54]. Furthermore, the enhanced fluorescence intensity in the wavelength range of 450–550 nm observed in the glucosamine group further supported the hypothesis that glucosamine facilitates the Maillard reaction more efficiently than other reducing sugars, generating a higher concentration of fluorescent intermediates [55].

### 3.5. Quantitative Sensory Evaluation of MRPs

**Figure 5:**
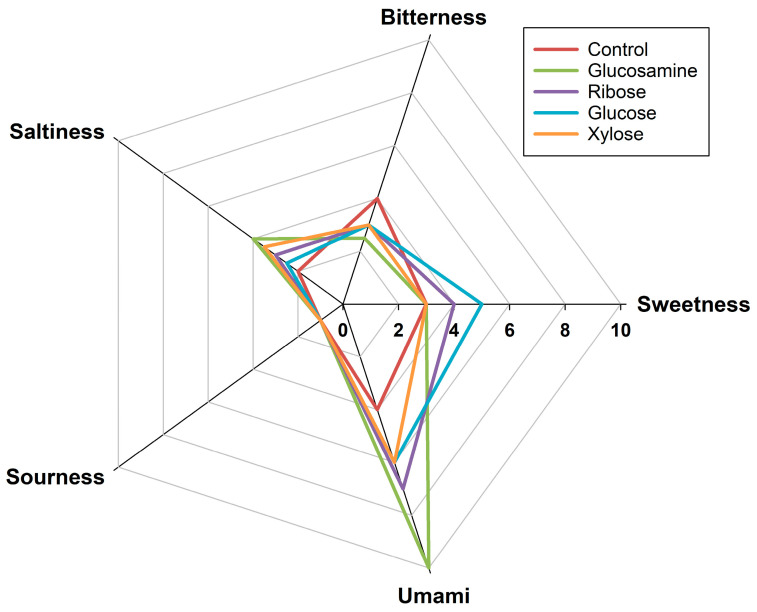
Radar chart of sensory evaluation of MRPs from different reducing sugars.

The sensory profiles of FSCPs without sugar and MRPs derived from xylose, glucosamine, glucose, and ribose were evaluated by a trained sensory panel. As shown in Figure 5, the bitterness intensity followed the order: native FSCPs (4.0) > ribose-MRPs (3.0) = glucose-MRPs (3.0) = xylose-MRPs (3.0) > glucosamine-MRPs (2.5). These results indicated that the Maillard reaction reduced the overall bitterness and fishy odor of FSCPs to varying degrees [56], with glucosamine-MRPs exhibiting the most significant reduction in bitterness compared to the control. For umami taste, the scores were glucosamine-MRPs (10.0) > ribose-MRPs (7.0) > glucose-MRPs (6.0) = xylose-MRPs (6.0) > FSCPs without sugar (4.0). The glucosamine-MRPs showed the highest umami intensity, suggesting that the sample not only has a more pronounced umami taste but also the strongest umami-enhancing effect [57]. Regarding saltiness, glucosamine-MRPs scored 4.0, followed by xylose-MRPs (3.5), ribose-MRPs (3.0), glucose-MRPs (2.5), and FSCPs without sugar (2.0). The increase in saltiness score from 2.0 (control) to 4.0 (glucosamine group) further confirmed the salt-enhancing capability of glucosamine-induced MRPs. However, no significant improvement was observed in sweetness and sourness across all groups.

The current findings were consistent with several previous reports. Zhou et al. demonstrated that MRPs could enhance umami while reducing fishy and bitter off-flavors in hydrolysates [57]. Recently, Zhang et al. have used xylose to mask the bitterness of defluorinated Antarctic krill hydrolysates by generating meaty flavor via the Maillard reaction under 105 °C [58]. In terms of umami enhancement, Ogasawara et al. [46] found that peptides with a MW of 1–5 kDa exhibited synergistic umami effects. This aligns with the results in Section 3.2, which revealed that glucosamine-MRPs contained a higher proportion of 1–5 kDa peptides (62.83%). These effects are attributed to the cross-linked products formed in the later stages of the Maillard reaction, especially between sugars and peptides. The smaller the MW of FSCPs, the more reactive they are with sugars, which facilitates the formation of flavor-enhancing glycopeptide conjugates during the Maillard reaction [59]. Regarding salt taste, Zhang et al., also reported that MRPs formed from collagen peptides and different reducing sugars showed variable salt-enhancing effects [60]. Among them, glucosamine-MRPs exhibited the most pronounced enhancement, increasing saltiness by 18.01% compared to 51 mmol/L NaCl solution [60]. The saltiness enhancement effect is believed to be mediated by Maillard reaction peptides (1–5 kDa) interacting with the transient receptor potential vanilloid 1 saltiness receptor, thereby producing a stronger perception of saltiness [53]. Overall, MRPs derived from glucosamine showed significantly reduced bitterness and enhanced umami and salt taste, demonstrating that Maillard reaction with glucosamine effectively improved the flavor profile of FSCPs by suppressing off-flavors and enhancing desirable taste.

### 3.6. Volatile Compound Analysis of MRPs

**Figure 6:**
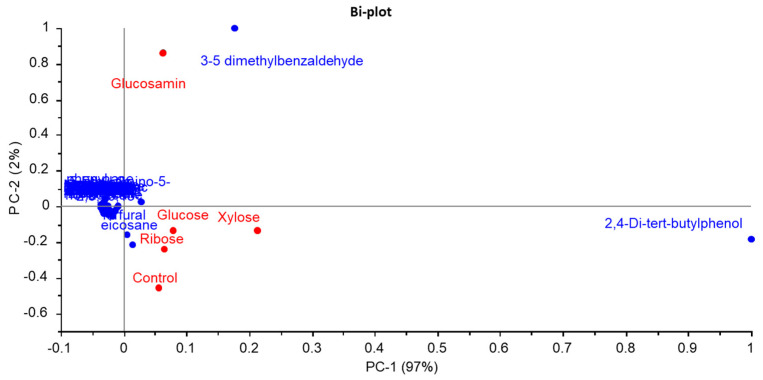
PCA bi−plot of volatile compounds from MRPs of different reducing sugars.

Principal component analysis (PCA) based on the GC-MS data of FSCPs and their MRPs is presented in Figure 6. The cumulative variance contribution of PC1 and PC2 reached 98%, indicating that these two PC plots captured most of the variation in volatile compounds among the samples. As shown in Figure 6, the glucosamine-derived MRPs were distinctly separated from both the FSCPs without sugar and the other three MRP groups, suggesting that glucosamine significantly altered the composition of volatile compounds. Among them, 3,5-dimethylbenzaldehyde was identified as a characteristic compound in the glucosamine group. This result aligned with findings by Cui et al., who used gas chromatography–ion mobility spectrometry to analyze flavor profiles in mushroom enzymatic hydrolysates undergoing Maillard reactions with various reducing agents, including xylose, fructose, glucose, sucrose, mannose, maltose, vitamin C, and L-arabinose [61]. A total of 41 volatile compounds were detected, and different sugar types led to distinct volatile profiles. Notably, the volatile composition of unreacted hydrolysates differed substantially from that of MRPs [61].

The observed PCA pattern, where the control showed proximity to the glucose, xylose, and ribose groups but was distinctly separated from the glucosamine-MRPs, offered insight into the reaction efficacy of different sugars. This contrasted with findings from Cui et al. [61], where the unreacted protein hydrolysate of *Lentinus edodes* (sample #8) was clearly separated from all MRPs prepared with various sugars. The discrepancy likely arises from differences in the peptide substrates and the extent of the Maillard reaction. In our study, the relatively mild Maillard reaction induced by glucose, xylose, and ribose under the given conditions (100 °C, 1 h) may not have generated a volatile profile substantially different from the control FSCPs. Glucosamine, however, owing to its high reactivity, promoted a more advanced Maillard reaction, leading to a significant transformation of the volatile compound profile and thus a clear separation in the PCA plot. This highlighted that the impact of Maillard reaction on flavor modification was highly dependent on both the reducing sugar type and the nature of protein hydrolysate.

GC-MS was used to analyze the volatile compounds in both FSCPs without sugar and MRPs. Quantification was performed using the peak area normalization method, and a total of 47 volatile compounds were identified across the samples, as shown in Table 2. The FSCPs without sugar contained the highest number of volatile compounds (29 types), while the number of volatile components decreased after Maillard reaction in all four reducing sugar groups. Among the volatile compounds identified in MRPs, phenolic and carbonyl compounds were predominant. In particular, 2,4-di-tert-butylphenol, which has a mild coconut aroma [62], may help mask fishy odors. Major aldehydes and ketones identified included 3,5-dimethylbenzaldehyde, phenylacetaldehyde, and furfural. Aldehydes are known as dominant volatiles in cooked pork products, imparting fruity, floral, and fatty notes [63]. Phenylacetaldehyde, generated via the Strecker degradation pathway, possesses a pleasant fruity aroma [64]. Notably, 3,5-dimethylbenzaldehyde, found in both FSCPs and all MRPs, contributed floral and fruity aromas [65]. Wang et al. identified 154 volatile compounds in MRPs derived from pufferfish muscle hydrolysates, with aldehydes comprising 23 of them [21]. Similarly, aldehydes were the most abundant volatile compounds in this study, particularly phenylacetaldehyde and 3-methylbutanal, both of which are Strecker aldehydes. As listed in Table 2, several undesirable volatile compounds decreased following the Maillard reaction, including n-hexadecane (fishy odor) [66], n-heptadecane (fishy odor) [66], eicosane (waxy odor) [67], 2,6,10,14-tetramethylpentadecane (fishy odor) [66], and di-isobutyl-phthalate (a harmful odorant) [68]. In contrast, aroma-enhancing compounds such as 3,5-dimethylbenzaldehyde (fruity odor) [65], 2,4-di-tert-butylphenol (coconut-like odor), and phenylacetaldehyde (fruity and almond-like odor) [69] showed a significant increase in content.

These changes in volatile compounds highlight the role of the Maillard reaction in improving flavor profile of FSCP. The reduction in undesirable fishy and waxy odors, such as n-hexadecane and eicosane, along with the increase in aroma-enhancing compounds like 3,5-dimethylbenzaldehyde and phenylacetaldehyde, contributed to a more favorable flavor profile. Additionally, 2,4-di-tert-butylphenol helps mask fishy odors, further improving the overall sensory characteristics. Statistical analysis of the data in Table 2 revealed significant differences (*p* < 0.05), with glucosamine-MRPs showing the most significant improvements in aroma-enhancing compounds, as listed in Table 2.

## 4. Conclusions

This study demonstrated that Maillard reaction modification with glucosamine most effectively enhanced the flavor profiles of FSCPs. Physicochemical analyses revealed that glucosamine-induced MRPs exhibited the greatest reduction in free amino groups (0.69 μmol/L), the most significant decrease in high-MW peptides (3.31%), and the highest color difference (Δ*E* = 31.78). Spectral analysis confirmed the intensified UV absorption (280–294 nm; 320–420 nm) and fluorescence intensity (400–550 nm), indicating abundant intermediate and fluorescent compound formation. Sensory evaluation showed glucosamine-treated MRPs achieved the most pronounced bitterness reduction alongside marked enhancements in umami and saltiness. GC-MS and PCA further verified that glucosamine MRPs minimize undesirable odorants (e.g., n-hexadecane, di-isobutyl-phthalate) while enriching key aroma compounds (e.g., 3,5-dimethylbenzaldehyde, phenylacetaldehyde). Collectively, the glucosamine-induced Maillard reaction optimally reduced off-flavors and promoted the formation of desirable sensory attributes, offering a strategic approach for flavor improvement in FSCPs. However, future research is still needed to further elucidate the mechanisms underlying glucosamine’s superior effect in the Maillard reaction for its potential applications in the food industry.

## Figures and Tables

**Table 1:** Color difference in MRPs from different reducing sugars.

Sample	L*	a*	b*	Δ*E*
Control	95.25 ± 0.57 ^a^	0.33 ± 0.04 ^c^	6.51 ± 0.08 ^d^	-
Glucosamine	85.70 ± 0.83 ^c^	2.55 ± 0.11 ^b^	36.74 ± 0.49 ^b^	31.78 ± 0.02 ^b^
Ribose	84.50 ± 0.57 ^c^	5.79 ± 0.07 ^a^	49.39 ± 0.53 ^a^	45.54 ± 0.08 ^a^
Glucose	95.23 ± 0.50 ^a^	0.34 ± 0.05 ^c^	6.68 ± 0.42 ^d^	0.17 ± 0.01 ^d^
Xylose	94.02 ± 0.62 ^ab^	0.02 ± 0.01 ^d^	13.19 ± 0.41 ^c^	6.80 ± 0.07 ^c^

Note: Values with different superscript letters in the same column indicate significant differences (*p* < 0.05).

**Table 2:** The relative content (%) of volatile compounds in different Maillard reaction products analyzed by GC-MS.

	SI	Compounds	Control (%)	Glucosamine	Ribose (%)	Glucose (%)	Xylose (%)
**Carbonyl compounds**	95	3,5-Dimethylbenzaldehyde	4.87 ± 0.11 ^d^	28.291 ± 0.21 ^a^	8.92 ± 0.20 ^b^	11.251 ± 0.26 ^b^	11.115 ± 0.24 ^b^
	88	7,9-Di-tert-butyl-1-oxaspiro [4.5]decane-6,9-diene-2,8-dione	0.903 ± 0.03 ^a^	0.849 ± 0.08 ^a^	0.57 ± 0.01 ^b^	0.718 ± 0.03 ^ab^	0.751 ± 0.05 ^ab^
	96	Benzaldehyde	-	1.221±0.05 ^a^	1.33±0.05 ^a^	-	-
	90	Isovaleraldehyde	-	-	3.41±0.06 ^a^	-	-
	95	Furfural	-	-	13.33±0.07 ^a^	-	-
**Alcohols**	93	2-Ethylhexanol	1.533 ± 0.03 ^a^	1.752 ± 0.02 ^a^	2.08 ± 0.06 ^a^	1.376 ± 0.05 ^a^	1.753 ± 0.08 ^a^
	95	Menthol	-	-	-	-	0.25 ± 0.04 ^a^
**Hydrocarbons**	83	Durene	0.393 ± 0.02^a^	-	-	-	-
	90	2,5-Dimethyl-Nonane	-	-	-	0.269 ± 0.02 ^a^	-
	95	2,6-Dimethylnonane	0.589 ± 0.04 ^b^	-	-	0.928 ± 0.05 ^a^	0.188 ± 0.01 ^c^
	93	4,6-Dimethylundecane	1.728 ± 0.12 ^a^	-	-	-	-
	87	2,3,5,8-Tetramyldecane	0.943 ± 0.05 ^a^	-	-	-	-
	96	Dodecane	0.275 ± 0.02 ^b^	0.372 ± 0.04 ^a^	-	0.18 ± 0.01 ^c^	0.157 ± 0.02 ^c^
	90	2,3,6-Trimethyldecane	0.118 ± 0.01 ^a^	-	-	-	-
	88	2,6,11-Trimethyl dodecane	0.860 ± 0.06 ^ab^	0.531 ± 0.02 ^c^	1.18 ± 0.02 ^a^	0.898 ± 0.04 ^ab^	0.877 ± 0.05 ^ab^
	93	Pentadecane	1.41 ± 0.03 ^a^	0.265 ± 0.03 ^b^	0.18 ± 0.02 ^b^	0.269 ± 0.07 ^b^	0.282 ± 0.06 ^b^
	88	Nonadecane	2.95 ± 0.04 ^a^	0.478 ± 0.03 ^b^	0.39 ± 0.03 ^b^	-	0.407 ± 0.05 ^b^
	91	2,7-Dimethylundecane	0.786 ± 0.07 ^a^	-	-	-	-
	92	Eicosane	12.22 ± 0.14 ^a^	1.592 ± 0.03 ^b^	0.54 ± 0.01 ^c^	1.346 ± 0.02 ^b^	1.659 ± 0.08 ^b^
	88	Heptadecane	0.314 ± 0.03 ^a^	-	-	-	-
	85	11-Methyltritadecane	0.314 ± 0.04 ^a^	-	-	-	-
	94	3-Methylpentadecane	0.353 ± 0.06 ^a^	-	-	-	-
	90	2,6,10,15-Tetracontane	2.99 ± 0.09 ^a^	0.584 ± 0.07 ^c^	0.50 ± 0.04 ^c^	1.197 ± 0.11 ^b^	0.595 ± 0.16 ^c^
	93	2,6,10-Trimethylpentadecane	0.471 ± 0.08 ^a^	-	-	-	-
	93	Hexadecane	0.825 ± 0.09 ^a^	0.69 ± 0.03^a^	-	0.509 ± 0.02 ^ab^	-
	91	2,6,10,14-Tetramethylpentadecane	1.65 ± 0.04 ^a^	-	-	0.269 ± 0.02 ^b^	-
	94	3-Methylheptadecane	0.196 ± 0.02 ^a^	0.053 ± 0.01 ^b^	-	0.09 ± 0.01 ^b^	-
	97	9-Docosene	3.692 ± 0.02 ^a^	2.07 ± 0.08 ^b^	0.61 ± 0.06 ^c^	0.868 ± 0.05 ^c^	1.19 ± 0.07 ^c^
	96	1,2,3-Trimethylbenzene	-	-	-	-	0.501 ± 0.01 ^a^
	92	3,7-Dimethyldecane	-	-	-	2.304 ± 0.06 ^a^	1.096 ± 0.05 ^a^
	92	2-Methyl octadecane	-	-	-	-	0.094 ± 0.01 ^a^
	90	3-Ethyl toluene	-	-	0.25 ± 0.02 ^b^	1.257 ± 0.05 ^a^	-
	92	5-Methyl tetradecane	-	-	0.11 ± 0.01 ^a^	0.15 ± 0.01 ^a^	-
	92	5-Ethyl-2-methyl octane	-	0.796 ± 0.02 ^a^	0.93 ± 0.03 ^a^	-	-
	92	5-(2-Methylpropyl) nonane	-	0.212 ± 0.03 ^a^	-	-	-
**Esters**	87	Isobutyric acid 3-hydroxy-2,2,4-trimethylpentyl ester	0.118 ± 0.02 ^a^	0.159 ± 0.01 ^a^	-	-	-
	90	2,2,4-Trimethyl-1,3-pentanediol di-isobutyrate	1.453 ± 0.03 ^a^	0.265 ± 0.03 ^b^	0.14 ± 0.01 ^b^	0.299 ± 0.02 ^b^	0.188 ± 0.01 ^b^
	94	Di-isobutyl phthalate	2.435 ± 0.01 ^a^	1.327 ± 0.01 ^b^	1.29 ± 0.01 ^b^	1.227 ± 0.01 ^b^	1.127 ± 0.01 ^b^
	90	Methyl silicate (petroleum-based)	0.746 ± 0.02 ^a^	-	-	-	-
	89	Methyl conjugated linoleic acid (9-cis,11-trans)	-	-	-	-	0.188 ± 0.01 ^a^
	90	Methyl oleate	-	-	0.39 ± 0.03 ^a^	-	0.344 ± 0.02 ^a^
	91	Methyl 10-octadecenoate	-	-	-	0.449 ± 0.03 ^a^	-
	88	Methyl 8-octadecenoate	-	0.212 ± 0.01 ^a^	-	-	-
	90	Methyl linoleate	-	-	0.18 ± 0.01 ^a^	-	-
**Acidic compounds**	82	2-Amino-5-methylbenzoic acid	2.396 ± 0.01 ^c^	3.875 ± 0.06 ^b^	4.37 ± 0.02 ^b^	2.573 ± 0.02 ^c^	5.917 ± 0.05 ^a^

Note: SI stands for similarity index; “-“ indicates below the detection limit. Different letters between each group indicate significant differences between treatments (*p* < 0.05).

## Data Availability

The original contributions presented in this study are included in the article. Further inquiries can be directed to the corresponding author.

## References

[1] Zhou Y., Zhang Y., Dai H., Zhang Y., Fu Y. (2024). The potential of undenatured type II collagen against arthritis: A review. Collagen Leather.

[2] Felician F.F., Xia C., Qi W., Xu H. (2018). Collagen from Marine Biological Sources and Medical Applications. Chem. Biodivers..

[3] Ahmed M., Verma A.K., Patel R. (2020). Collagen extraction and recent biological activities of collagen peptides derived from sea-food waste: A review. Sustain. Chem. Pharm..

[4] Grand View R. (2024). *Collagen Market Size, Share & Growth|Industry Report 2030*; Grand View Research. https://www.grandviewresearch.com/industry-analysis/collagen-market.

[5] Future Market I. (2025). Collagen Peptide Market Size, Trends & Forecast 2025–2035.

[6] Nurilmala M., Hizbullah H.H., Karnia E., Kusumaningtyas E., Ochiai Y. (2020). Characterization and Antioxidant Activity of Collagen, Gelatin, and the Derived Peptides from Yellowfin Tuna (Thunnus albacares) Skin. Mar. Drugs.

[7] Nurilmala M., Suryamarevita H., Husein Hizbullah H., Jacoeb A.M., Ochiai Y. (2022). Fish skin as a biomaterial for halal collagen and gelatin. Saudi J. Biol. Sci..

[8] Ge B., Wang H., Li J., Liu H., Yin Y., Zhang N., Qin S. (2020). Comprehensive Assessment of Nile Tilapia Skin (Oreochromis niloticus) Collagen Hydrogels for Wound Dressings. Mar. Drugs.

[9] Luo J., Zhou Z., Yao X., Fu Y. (2020). Mineral-chelating peptides derived from fish collagen: Preparation, bioactivity and bioavailability. LWT.

[10] Thirukumaran R., Anu Priya V.K., Krishnamoorthy S., Ramakrishnan P., Moses J.A., Anandharamakrishnan C. (2022). Resource recovery from fish waste: Prospects and the usage of intensified extraction technologies. Chemosphere.

[11] Lee J.K., Jeon J.-K., Byun H.-G. (2014). Antihypertensive effect of novel angiotensin I converting enzyme inhibitory peptide from chum salmon (Oncorhynchus keta) skin in spontaneously hypertensive rats. J. Funct. Foods.

[12] Li X., Liu Y., Wang Y., Wang J., Xu Y., Yi S., Zhu W., Mi H., Li T., Li J. (2021). Combined ultrasound and heat pretreatment improve the enzymatic hydrolysis of clam (Aloididae aloidi) and the flavor of hydrolysates. Innov. Food Sci. Emerg. Technol..

[13] Liu Y., Huang Y., Wang Z., Cai S., Zhu B., Dong X. (2021). Recent advances in fishy odour in aquatic fish products, from formation to control. Int. J. Food Sci. Technol..

[14] Mohammadi M., Mirza Alizadeh A., Mollakhalili N. (2021). Off-Flavors in Fish: A Review of Potential Development Mechanisms, Identification and Prevention Methods. J. Hum. Environ. Health Promot..

[15] Lindholm-Lehto P.C., Vielma J., Pakkanen H., Alén R. (2019). Depuration of geosmin- and 2-methylisoborneol-induced off-flavors in recirculating aquaculture system (RAS) farmed European whitefish Coregonus lavaretus. J. Food Sci. Technol..

[16] Ruan Q., An Y., Chen Z., You J., Xiong S. (2021). Effect of short time micro water treatment on fish flavor quality of grass carp. J. Food Sci. Technol..

[17] Liu Y., Duan Z., Cai Y., Hu B. (2015). Study on the defishment process of oyster enzymatic hydrolysate. Food Res. Dev..

[18] Xiong H., Cao L., Yan Q., Ma Y. (2013). Study on optimization of the Maillard reaction process of Tilapia fish platoon protease hydrolysate. Chin. Agric. Sci. Bull..

[19] Li Y., Zhou W., Cao Y., Gong X., Li J., Lu X., Dai Y. (2020). Analysis of volatile components of Tilapia enzymolysis solution after different deodorization treatments. IOP Conf. Ser. Earth Environ. Sci..

[20] Hu Y., Shi W., Lu Y. (2021). Research progress of fish odor removal technology. Food Ferment. Ind..

[21] Wang W., Zhang L., Wang Z., Wang X., Liu Y. (2019). Physicochemical and sensory variables of Maillard reaction products obtained from Takifugu obscurus muscle hydrolysates. Food Chem..

[22] Li Y., Wang X., Xue Y., Ruan S., Zhou A., Huang S., Ma H. (2021). The Preparation and Identification of Characteristic Flavour Compounds of Maillard Reaction Products of Protein Hydrolysate from Grass Carp (Ctenopharyngodon idella) Bone. J. Food Qual..

[23] Fu Y., Zhang Y., Soladoye O.P., Aluko R.E. (2020). Maillard reaction products derived from food protein-derived peptides: Insights into flavor and bioactivity. Crit. Rev. Food Sci. Nutr..

[24] Kouakou C., Bergé J.-P., Baron R., Lethuaut L., Prost C., Cardinal M. (2014). Odor Modification in Salmon Hydrolysates Using the Maillard Reaction. J. Aquat. Food Prod. Technol..

[25] Vhangani L.N., Van Wyk J. (2013). Antioxidant activity of Maillard reaction products (MRPs) derived from fructose–lysine and ribose–lysine model systems. Food Chem..

[26] Khadidja L., Asma C., Mahmoud B., Meriem E. (2017). Alginate/gelatin crosslinked system through Maillard reaction: Preparation, characterization and biological properties. Polym. Bull..

[27] Maillard M.N., Billaud C., Chow Y.N., Ordonaud C., Nicolas J. (2007). Free radical scavenging, inhibition of polyphenoloxidase activity and copper chelating properties of model Maillard systems. LWT—Food Sci. Technol..

[28] Karbasi M., Madadlou A. (2018). Interface-related attributes of the Maillard reaction-born glycoproteins. Crit. Rev. Food Sci. Nutr..

[29] Fu Y., Liu J., Zhang W., Wæhrens S.S., Tøstesen M., Hansen E.T., Bredie W.L.P., Lametsch R. (2020). Exopeptidase treatment combined with Maillard reaction modification of protein hydrolysates derived from porcine muscle and plasma: Structure–taste relationship. Food Chem..

[30] Hemmler D., Roullier-Gall C., Marshall J.W., Rychlik M., Taylor A.J., Schmitt-Kopplin P. (2018). Insights into the Chemistry of Non-Enzymatic Browning Reactions in Different Ribose-Amino Acid Model Systems. Sci. Rep..

[31] Song C., Yang Y., Zhao Z., Tan M., Chen Z., Zheng H., Gao J., Lin H., Zhu G., Cao W. (2024). Insight into the correlation of taste substances and salty-umami taste from Monetaria moneta hydrolysates prepared using different proteases. Food Chem. X.

[32] Adler-Nissen J., Olsen H.S. (1979). The Influence of Peptide Chain Length on Taste and Functional Properties of Enzymatically Modified Soy Protein.

[33] Fu Y., Liu J., Hansen E.T., Bredie W.L.P., Lametsch R. (2018). Structural characteristics of low bitter and high umami protein hydrolysates prepared from bovine muscle and porcine plasma. Food Chem..

[34] Fu Y., Bak K.H., Liu J., De Gobba C., Tøstesen M., Hansen E.T., Petersen M.A., Ruiz-Carrascal J., Bredie W.L.P., Lametsch R. (2019). Protein hydrolysates of porcine hemoglobin and blood: Peptide characteristics in relation to taste attributes and formation of volatile compounds. Food Res. Int..

[35] Gomide A.I., Silva R., Nascimento M., Minim L.A., Minim V.P.R. (2021). Study of the influence of line scale length (9 and 15 cm) on the sensory evaluations of two descriptive methods. J. Food Sci. Technol..

[36] Yang Y., Wang B., Fu Y., Shi Y.-G., Chen F.-L., Guan H.-N., Liu L.-L., Zhang C.-Y., Zhu P.-Y., Liu Y. (2021). HS-GC-IMS with PCA to analyze volatile flavor compounds across different production stages of fermented soybean whey tofu. Food Chem..

[37] Chiang J.H., Eyres G.T., Silcock P.J., Hardacre A.K., Parker M.E. (2019). Changes in the physicochemical properties and flavour compounds of beef bone hydrolysates after Maillard reaction. Food Res. Int..

[38] Sun A., Chen L., Wu W., Soladoye O.P., Zhang Y., Fu Y. (2023). The potential meat flavoring generated from Maillard reaction products of wheat gluten protein hydrolysates-xylose: Impacts of different thermal treatment temperatures on flavor. Food Res. Int..

[39] Qin Z., Han Y.-F., Wang N.-N., Liu H.-M., Zheng Y.-Z., Wang X.-D. (2020). Improvement of the oxidative stability of cold-pressed sesame oil using products from the Maillard reaction of sesame enzymatically hydrolyzed protein and reducing sugars. J. Sci. Food Agric..

[40] Yu B., Gong X., Zhang N., Benjakul S., Zhang Y., Fu Y. (2024). Glycation modification of protein hydrolysate from channel catfish (Ictalurus Punetaus) viscera to mitigate undesirable flavor: Unraveling structure and flavor characteristics. Food Chem. X.

[41] Zhang Y., Zhang Z., Fu Y., Gao Y., Guo W., Hu R., Liu X. (2023). Effects of different ph on properties of heat-induced Auricularia auricula-judae polysaccharide-whey protein isolate composite gels. Food Struct..

[42] YU A.-N., TANG L.-P. (2016). Kinetics of non-enzymatic browning reaction from the l-ascorbic acid/l-cysteine model system. Czech J. Food Sci..

[43] Hong P.K., Ndagijimana M., Betti M. (2016). Glucosamine-induced glycation of hydrolysed meat proteins in the presence or absence of transglutaminase: Chemical modifications and taste-enhancing activity. Food Chem..

[44] Chen Y., Zhang M., Mujumdar A.S., Liu Y. (2022). Combination of epigallocatechin gallate with l-cysteine in inhibiting Maillard browning of concentrated orange juice during storage. LWT.

[45] Shen Y., Hu L.T., Xia B., Ni Z.J., Elam E., Thakur K., Zhang J.G., Wei Z.J. (2021). Effects of different sulfur-containing substances on the structural and flavor properties of defatted sesame seed meal derived Maillard reaction products. Food Chem..

[46] Ogutu B., Kim Y.J., Kim D.W., Oh S.C., Hong D.L., Lee Y.B. (2017). Optimization of Maillard Reaction between Glucosamine and Other Precursors by Measuring Browning with a Spectrophotometer. Prev. Nutr. Food Sci..

[47] Wei C.-K., Ni Z.-J., Thakur K., Liao A.-M., Huang J.-H., Wei Z.-J. (2019). Color and flavor of flaxseed protein hydrolysates Maillard reaction products: Effect of cysteine, initial pH, and thermal treatment. Int. J. Food Prop..

[48] Giovanelli G., Cappa C. (2021). 5-Hydroxymethylfurfural Formation in Bread as a Function of Heat Treatment Intensity: Correlations with Browning Indices. Foods.

[49] Cao C., Xie J., Hou L., Zhao J., Chen F., Xiao Q., Zhao M., Fan M. (2017). Effect of glycine on reaction of cysteine-xylose: Insights on initial Maillard stage intermediates to develop meat flavor. Food Res. Int..

[50] Fu B., Xu X., Zhang X., Cheng S., El-Seedi H.R., Du M. (2023). Identification and characterisation of taste-enhancing peptides from oysters (Crassostrea gigas) via the Maillard reaction. Food Chem..

[51] Sun L., Zhuang Y. (2012). Characterization of the Maillard Reaction of Enzyme-Hydrolyzed Wheat Protein Producing Meaty Aromas. Food Bioprocess Technol..

[52] Hrynets Y., Ndagijimana M., Betti M. (2015). Studies on the Formation of Maillard and Caramelization Products from Glucosamine Incubated at 37 °C. J. Agric. Food Chem..

[53] Yu B., Wu W., Wang B., Zhang N., Bak K.H., Soladoye O.P., Aluko R.E., Zhang Y., Fu Y. (2022). Maillard-reacted peptides from glucosamine-induced glycation exhibit a pronounced salt taste-enhancing effect. Food Chem..

[54] Li X., Yao Y., Xia X., Zhang F., Yu J., Cui H., Niu Y., Hayat K., Zhang X., Ho C.-T. (2024). Maillard Reaction Process and Characteristic Volatile Compounds Formed During Secondary Thermal Degradation Monitored via the Change of Fluorescent Compounds in the Reaction of Xylose–Corn Protein Hydrolysate. J. Agric. Food Chem..

[55] Qiu J., Li H., Liu Y., Li C., Fang Z., Hu B., Li X., Zeng Z., Liu Y. (2024). Changes in flavor and biological activities of Lentinula edodes hydrolysates after Maillard reaction. Food Chem..

[56] Bak K.H., Waehrens S.S., Fu Y., Chow C.Y., Petersen M.A., Ruiz-Carrascal J., Bredie W.L.P., Lametsch R. (2021). Flavor Characterization of Animal Hydrolysates and Potential of Glucosamine in Flavor Modulation. Foods.

[57] Zhou X., Cui H., Zhang Q., Hayat K., Yu J., Hussain S., Tahir M.U., Zhang X., Ho C.-T. (2021). Taste improvement of Maillard reaction intermediates derived from enzymatic hydrolysates of pea protein. Food Res. Int..

[58] Zhang D., Ji W., Peng Y., Ji H., Gao J. (2020). Evaluation of Flavor Improvement in Antarctic Krill Defluoridated Hydrolysate by Maillard Reaction Using Sensory Analysis, E-nose, and GC-MS. J. Aquat. Food Prod. Technol..

[59] Karangwa E., Murekatete N., Habimana Jde D., Masamba K., Duhoranimana E., Muhoza B., Zhang X. (2016). Contribution of crosslinking products in the flavour enhancer processing: The new concept of Maillard peptide in sensory characteristics of Maillard reaction systems. J. Food Sci. Technol..

[60] Zhang T. (2021). Effects of four reducing sugars on physicochemical properties and saltiness-enhancing ability of Maillard reaction products derived from fish skin collagen peptides. Food Ferment. Ind..

[61] Cui W., Liu P., Liu H., Sun Y., Wang W. (2023). Flavor changes in Lentinus Edodes enzymatic hydrolysate maillard reaction products with different sugars by gas chromatography-ion mobility spectrometry. J. Future Foods.

[62] Zhang D., Sun G., Zhang X. (2019). Analysis of a Gas Chromatography/Mass Spectrometry Thermal Desorption System with Simultaneous Sniffing for Determination of Off-Odor Compounds and Volatile Organic Compounds in Polypropylene Composites. Sci. Adv. Mater..

[63] Liu H., Hui T., Fang F., Ma Q., Li S., Zhang D., Wang Z. (2021). Characterization and Discrimination of Key Aroma Compounds in Pre- and Postrigor Roasted Mutton by GC-O-MS, GC E-Nose and Aroma Recombination Experiments. Foods.

[64] Tian L.L., Han F., Fodjo E.K., Zhai W., Huang X.Y., Kong C., Shi Y.F., Cai Y.Q. (2021). An Effective and Efficient Sample Preparation Method for 2-Methyl-Isoborneol and Geosmin in Fish and Their Analysis by Gas Chromatography-Mass Spectrometry. Int. J. Anal. Chem..

[65] Zhang W., Lao F., Bi S., Pan X., Pang X., Hu X., Liao X., Wu J. (2021). Insights into the major aroma-active compounds in clear red raspberry juice (*Rubus idaeus* L. cv. Heritage) by molecular sensory science approaches. Food Chem..

[66] Cai L., Cao M., Cao A., Zhang W. (2019). The Effect of Magnetic Nanoparticles Plus Microwave Thawing on the Volatile Flavor Characteristics of Largemouth Bass (*Micropterus salmoides*) Fillets. Food Bioprocess Technol..

[67] Munir M., Nadeem M., Ali B., Sultan M., Kanwal R., Al-Jumayi H.A., Algarni E.H.A., Alnofeai M.B., Mahmoud S.F. (2022). Investigating the Impact of Ultrasound, Microwave, and High-Pressure Processing of Milk on the Volatile Compounds and Sensory Properties of Cheddar Cheese. Agriculture.

[68] Buyukada M. (2019). Removal, potential reaction pathways, and overall cost analysis of various pollution parameters and toxic odor compounds from the effluents of turkey processing plant using TiO_2_–assisted UV/O_3_ process. J. Environ. Manag..

[69] Qian M., Zheng M., Zhao W., Liu Q., Zeng X., Bai W. (2021). Effect of marinating and frying on the flavor of braised pigeon. J. Food Process. Preserv..

